# Single Purse-String Suture for Reinforcement of Duodenal Stump During Laparoscopic Radical Gastrectomy for Gastric Cancer

**DOI:** 10.3389/fonc.2019.01020

**Published:** 2019-10-09

**Authors:** Hongyong He, Haojie Li, Botian Ye, Fenglin Liu

**Affiliations:** Department of General Surgery, Zhongshan Hospital, Fudan University, Shanghai, China

**Keywords:** gastric cancer, laparoscopic radical gastrectomy, duodenal stump leakage, laparoscopic single purse-string suture, reinforcement

## Abstract

**Background:** Duodenal stump leakage (DSL) is a serious surgical complication after radical gastrectomy with Roux-en-Y or BillrothII reconstruction. This study was designed to evaluate the effectiveness of laparoscopic single purse-string suture for reinforcement of duodenal stump.

**Methods:** A total of 183 patients harboring gastric adenocarcinoma following laparoscopic radical gastrectomy with Roux-en-Y or BillrothIIreconstruction and single purse-string suture for reinforcement of duodenal stump were retrospectively enrolled from Zhongshan Hospital of Fudan University (Shanghai, China) between January 2014 and December 2016. Operative variables and short-term complications were documented and analyzed. Clavien-Dindo classification system was used to identify surgical complications.

**Results:** Among 183 patients, 108 (59.02%) patients received distal gastrectomy and 75 (40.98%) received total gastrectomy. 88 (48.09%) patients underwent Roux-en-Y reconstruction and 95 (51.91%) patients underwent Billroth-II reconstruction. The mean time of laparoscopic single purse-string suture was 5.01 ± 1.33 min (range from 3.6 to 10.2 min). Postoperative early complication occurred in 26 cases of the patients. There were 4 cases of system-related complications (2.19%), including 3 cases of pulmonary infection (1.64%) and 1 cases of cardiovascular event (0.55%); and 22 cases of surgery-related complications (12.02%), including 6 cases of intra-abdominal infection (3.28%), 4 cases of pancreatic leakage (2.19%), 4 cases of wound complications (2.19%), 3 cases of gastroparesis (1.64%), 2 cases of intra-abdominal bleeding (1.09%), 2 cases of ileus (1.09%), 1 cases of lymphatic leakage (0.55%), and no duodenal stump leakage.

**Conclusion:** Reinforcement on duodenal stump using laparoscopic single purse-string suture during laparoscopic radical gastrectomy is simple and effective and could avoid the incidence of duodenal stump leakage to some extent.

## Introduction

Duodenal stump leakage (DSL) is serious surgical complication following radical gastrectomy with Roux-en-Y or BillrothII reconstruction (1). It is very hard to treat and is fatal in some cases (2–4). Factors associated with DSL can be divided into systemic factors and local factors (5, 6). Age, nutritional status, comorbidities were considered as systemic factors associated with DSL (5). The local factors, such as excessive vascular dissection around duodenal stump and direct thermal injury, might influence the healing of duodenal stump and result in DSL (6). In addition, DSL can also be associated with high pressure in the cavity of duodenal stump due to afferent loop obstruction or acute pancreatitis (7, 8).

Traditionally, surgeons may choose interrupted or continuous sutures to reinforce the duodenal stump in open gastrectomy (1, 8). While, it is relatively more difficult for most unexperienced surgeons to manually perform it during laparoscopic surgery as sophisticatedly as open surgery. Based on this practical problem, and the incidence of DSL is not very high, some surgeons proposed their view that duodenal stump do not need to be reinforced in laparoscopic gastrectomy. However, the consequences of DSL are very serious. It is necessary to develop a simple and effective method to reinforce the duodenal stump and release the pressure at the edge of the duodenal stump during laparoscopic surgery.

In the current study, we introduced a new and simple maneuver, single purse-string suture, for laparoscopic reinforcement of duodenal stump, which could be done by the surgeon alone easily and avoid the incidence of DSL to some extent.

## Methods

### Patients

We prospectively recruited consecutive patients with gastric cancer, collected the clinicopathological data, and detailed retrospectively analyzed the clinicopathological features correlating with morbidity and mortality and their role in decreasing the incidence of complications and the death rate, and improving the effect of operation (9). Between January 2014 and December 2016, a total of 183 patients harboring gastric adenocarcinoma following laparoscopic radical gastrectomy with Roux-en-Y or Billroth II reconstruction and single purse-string suture for reinforcement of duodenal stump were retrospectively enrolled from Zhongshan Hospital of Fudan University (Shanghai, China). Excluded were patients with distant metastases, gastric stump cancer, and peritoneal dissemination. In addition, patients were excluded if they had previously been exposed to any chemotherapy, radiotherapy, targeted therapy, or intervention therapy for gastric cancer. A retrospective review of prospectively collected data was performed, and the clinicopathological features (patient's age, gender, tumor localization, co-morbidity, tumor size, history of abdominal surgery, depth of tumor invasion, lymphatic vessel invasion, distant metastases, and pathological TNM stage) and the operation results (morbidity and mortality) were analyzed. The stage of gastric cancer is classified according to the tumor-node-metastasis (TNM) staging system of the eighth UICC/AJCC manual (10). The postoperative complications are defined and graded according to the grading system of Clavien-Dindo classification (11).

### Surgical Procedure

Patients were placed in a modified reverse trendelenburg position with the head slightly elevated. The primary operator stood on the left side of the patient, the first assistant was on the opposite side and the camera assistant stood between the legs of the patient.

During the port placement process, a 1–1.5 cm curved incision was made just below the umbilicus for a 10-mm trocar. After establishing pneumoperitoneum at 12 mmHg, the camera was inserted and the diagnostic laparoscopy was performed. The major operative port was placed in the left upper quadrant at the crossing of mid-clavicle line and arc of rib with a 12 mm trocar, and another trocar of 5 mm was inserted in the left lower quadrant at the crossing of mid-clavicle line and umbilical horizon. Two additional ports were placed in the right upper and right lower quadrant, both with 5 mm trocars, for the first assistant's instruments. The process of port placement could be adjusted according to the body shape of the patient and operator's preference.

Depending on the location of the tumor, the proximal free margin was at least 3 cm of esophagus for total gastrectomy and at least 5 cm for advanced tumors for distal gastrectomy. R0 resection and standard D1+/D2 lymphadenectomy was performed according to guideline of Japanese Gastric Cancer Association. Roux-en-Y and Billroth II reconstruction was performed in laparoscopic distal gastrectomy according to the size of residual stomach and operator's choice.

### Laparoscopic Duodenal Stump Reinforcement

**Figure 1:**
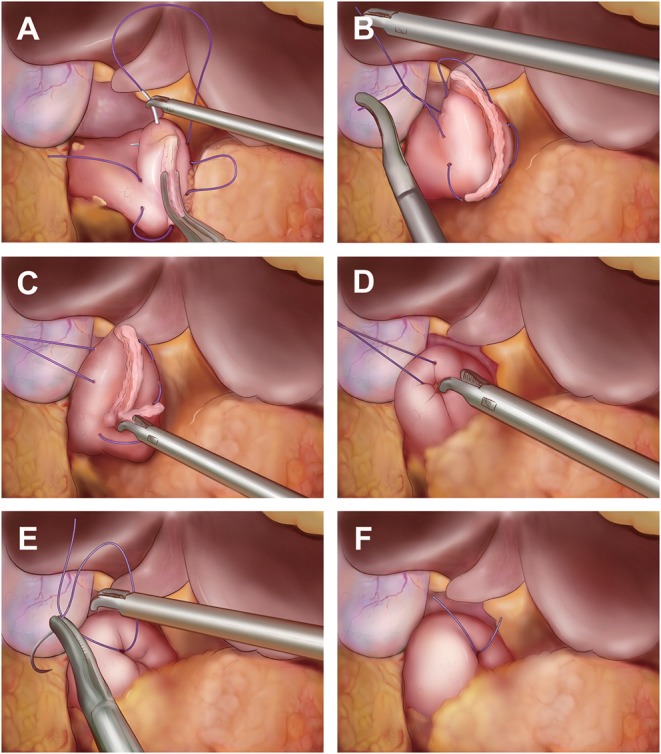
Reinforcement on duodenal stump using laparoscopic single purse-string suture. **(A)** Place a seromuscular purse-string suture on the duodenum wall 1.0–1.5 cm away from the duodenal stump using 3–0 single-strand absorbable suture; **(B)** Place a knot before tightening the purse-string suture; **(C)** Push the duodenal stump into the purse-string suture using laparoscopic needle holding or grasping forceps; **(D)** Tighten the knot after the duodenal stump into the purse-string suture totally; **(E)** Reinforce the knots of purse-string suture with 4–5 knots; **(F)** The photo of reinforcement finished.

Before dissecting the duodenum, approximately 2–3 cm of dissociated duodenum stump was preserved for reinforcement. A 60 mm endoscopic linear cutter (staple height 1.5–1.8 mm) was used to cut the duodenum from left side to right side. After cutting of duodenal stump, reinforcement on duodenal stump using laparoscopic single purse-string suture was performed as follows (Figure 1): a. place a seromuscular purse-string suture on the duodenum wall 1.0–1.5 cm away from the duodenal stump using 3-0 single-strand absorbable suture; b. place a knot before tightening the purse-string suture; c. push the duodenal stump into the purse-string suture using laparoscopic needle holding or grasping forceps; d. tighten the knot of the purse-string suture and reinforce it with 4–5 knots.

### Duodenal Stump Leakage

Duodenal stump leakage (DSL) was defined by the presence of bile in the drainage tube, which was placed near the duodenal stump during the operation; or there was regional or diffuse fluid collection near the duodenal stump and confirmed by an abdominal CT scan, which was performed in patients who represented symptoms of clinical suspects of DSL, such as severe and abrupt abdominal pain, fever, worsening leukocytosis, and so on.

### Statistical Analysis

The data were presented as mean ± standard deviation for continuous variables and as numbers and percentages for categorical variables. All analyses were performed using SPSS software (version 20.0, SPSS Inc., Chicago, IL, USA).

## Results

### Clinicopathological Features

The clinical and pathological characteristics were summarized in Table 1. The mean age of the patients was 54.25 ± 9.27 ys (range from 24 to 87 ys). Most patents were male (122 of 183, 66.67%), and 66 (36.07%) patients had co-morbidity, of which, hypertension (34 of 183, 18.58%) ranked the highest. The mean preoperative blood albumin was 41.31 ± 3.88 g/L (range from 28 to 51 g/L). 9 (4.92%) patients presented with history of abdominal surgery, including cholecystectomy, appendectomy, and others. More than a half (121 of 183, 66.12%) of patients presented with TNM stage I gastric cancer, no lymph node metastasis (123 of 183, 67.21%), and poorly differentiation (101 of 183, 55.19%).

**Table 1:** Patient demographics and clinicopathological characteristics.

**Factor**	**No. of patients**	**%**	**Mean**	**SD**
All patients	183	100		
Age(years)			54.25	9.27
Preoperative blood albumin, g/L			41.31	3.88
Preoperative blood creatinine, mmol/L			74.68	17.77
**Gender**				
Female	61	33.33		
Male	122	66.67		
**Localization**				
Proximal	53	28.96		
Middle	59	32.24		
Distal	71	38.80		
**Co-morbidity**				
Hypertension	34	18.58		
Diabetes mellitus	19	10.38		
Cardiac	13	7.10		
Tumor size (cm)			2.76	1.56
**Differentiation**				
Well	10	5.46		
Moderate	72	39.34		
Poorly	101	55.19		
**History of abdominal surgery**				
Cholecystectomy	1	0.55		
Appendectomy	5	2.73		
Others	3	1.64		
**Pathological T Stage**				
T1a	53	28.96		
T1b	51	27.87		
T2	39	21.31		
T3	24	13.11		
T4a	16	8.74		
T4b	0	0		
**Pathological *N* Stage**				
N0	123	67.21		
N1	21	11.48		
N2	25	13.66		
N3a	11	6.01		
N3b	3	1.64		
**Pathological M Stage**				
M0	183	100		
M1	0	0		
**Pathological TNM Stage**				
IA	93	50.82		
IB	28	15.30		
IIA	15	8.20		
IIB	31	16.94		
IIIA	8	4.37		
IIIB	5	2.73		
IIIC	3	1.64		
IV	0	0		

### Surgical Outcomes

Table 2 summarizes the surgical outcomes. Distal gastrectomy was performed in 108 (59.02%) patients and total gastrectomy was performed in 75 (40.98%) patients. Billroth II reconstruction was performed in 95 (51.91%) patients, Roux-en-Y reconstruction for 88 (48.09%) patients. The mean surgical time was 238.02 ± 53.07 min (range from 178 to 314 min). The procedure of laparoscopic single purse-string suture took 5.01 ± 1.33 min (range from 3.6 to 10.2 min). 37.83 ± 14.35 (range from 17 to 98) lymph nodes were retrieved from the patients enrolled in this study. There were 29 combined surgeries, including 27 cases of cholecystectomy, 1 case of splenectomy, and 1 case of adrenalectomy. Mean postoperative hospital stay was 9.82 ± 6.81 days (range from 5 to 50 days).

**Table 2:** Surgical outcomes.

**Outcome**	**No. of Patients**	**%**	**Mean**	**SD**
All patients	183	100		
**Extent of resection**				
Distal gastrectomy	108	59.02		
Total gastrectomy	75	40.98		
**Reconstruction**				
Billroth-II	95	51.91		
Roux-en-Y	88	48.09		
**Lymphadenectomy**				
D1+	41	22.40		
D2	142	77.60		
**Combined resection**				
Gallbladder	27	14.75		
Spleen	1	0.55		
Adrenal gland	1	0.55		
Retrieved lymph node			37.83	14.35
Embedding time, minutes			5.01	1.33
Estimated blood loss, mL			136.52	86.95
Surgical time, minutes			238.02	53.07
Postoperative hospital stay, days			9.82	6.81

### Morbidity and Mortality

In all, postoperative early complication occurred in 26 cases of the patients and no patient died (Table 3). There were 4 cases of system-related complications (2.19%), including 3 cases of pulmonary infection (1.64%) and 1 cases of cardiovascular event (0.55%); and 22 cases of surgery-related complications (12.02%), including 6 cases of intra-abdominal infection (3.28%), 4 cases of pancreatic leakage (2.19%), 4 cases of wound complications (2.19%), 3 cases of gastroparesis (1.64%), 2 cases of intra-abdominal bleeding (1.09%), 2 cases of ileus (1.09%), 1 cases of lymphatic leakage (0.55%), and no duodenal stump leakage. According to Clavien-Dindo classification, 23 patients were classified as ≤ II and 2 patients as IIIa. Only one case of intestinal obstruction recovered after reoperation, and patients with other complications were discharged successfully after conservative treatment.

**Table 3:** Morbidity and mortality.

**Morbidity type/Mortality**	**No. of Patients**	**%**
Morbidity	26	14.21
Surgery-related complications	22	12.02
Intra-abdominal infection	6	3.28
Pancreatic leakage	4	2.19
Wound complications	4	2.19
Gastroparesis	3	1.64
Intra-abdominal bleeding	2	1.09
Ileus	2	1.09
Lymphatic leakage	1	0.55
Duodenal stump leakage	0	0.00
System-related complications	4	2.19
Pulmonary infection	3	1.64
Cardiovascular event	1	0.55
Mortality	0	0.00
**Clavien-Dindo Classification**		
I	2	1.09
II	21	11.48
IIIa	2	1.09
IIIb	1	0.55

### Potential Mechanism

**Figure 2:**
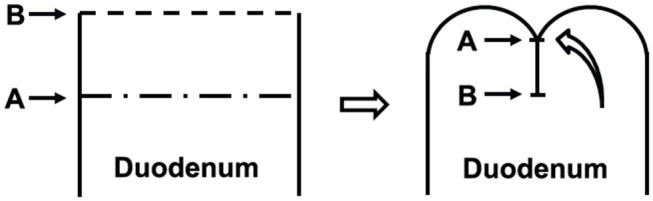
The pattern of the reinforcement of duodenal stump with single purse-string suture. The reinforcement was performed on the relatively normal tissue, which was the field to take the pressure of duodenum (point A). The staple-line of duodenal stump was the weak point (point B), which was protected and not need to take the pressure in the duodenum after the reinforcement.

As shown in Figure 2, there are two potential mechanisms of the avoidance of DSL after single purse-string suture for reinforcement of duodenal stump. First, the reinforcement was performed on the relatively normal tissue in contrast to other methods, such as barbed suture and Lembert suture, which are performed on the staple-line of duodenal stump directly; Second, the field of single purse-string suture (point A) is the force-bearing point, and the staple-line of duodenal stump (weak point, point B) has been protected. Above all, the field to take the pressure of duodenum (point A) is the relatively normal tissue, and the weak point (point B) is protected and not need to take the pressure in the duodenum, especially when the afferent loop obstruction occurred, so this maneuver could avoid the incidence of DSL effectively.

### Case Presentation

**Figure 3:**
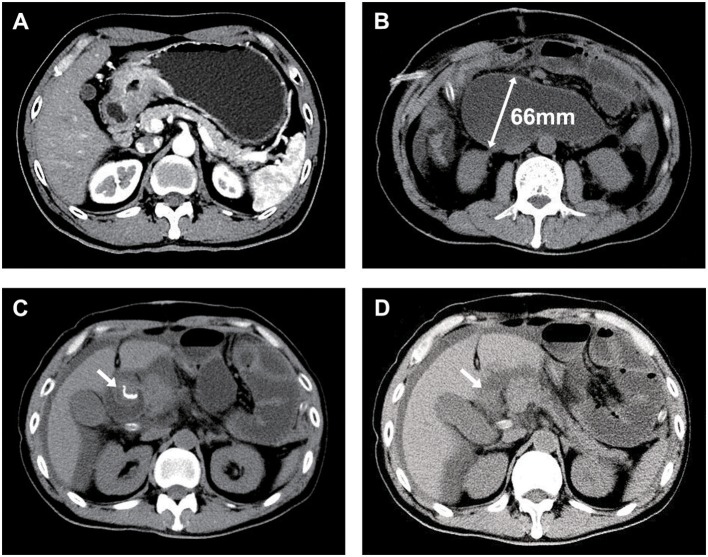
One case of afferent loop obstruction after Billroth II reconstruction and single purse-string suture for reinforcement of duodenal stump. **(A)** Abdominal CT image of the case with adenocarcinoma of gastric antrum; **(B)** After the operation, the afferent loop obstruction occurred, and the diameter of duodenum was more than 6 cm; **(C,D)** The reinforced duodenal stump (arrows) was intact.

In Dec. 2015, a 52-year-old man with adenocarcinoma of gastric antrum was referred to our institution and had laparoscopic assisted radical distal gastrectomy with Billroth II reconstruction and single purse-string suture for reinforcement of duodenal stump. After the operation, the afferent loop obstruction occurred, and the diameter of duodenum was more than 6 cm. However, we found the duodenal stump was intact according to the image of CT scan and confirmed it during our second operation (Figure 3). This case showed that single purse-string suture can withstand huge pressure in the duodenum.

## Discussion

Duodenal stump leakage (DSL) is severe complication with a high mortality rate after radical gastrectomy with Roux-en-Y or BillrothIIreconstruction, and the incidence rate is ranging from 1.6% to 5% (1, 2). Once DSL occurred, it is very difficult to treat and the mortality rate is reported as high as 16% to 20% (8). Patient age, nutritional status, comorbidities were considered as risk system factors associated with DSL after gastrectomy (5). In addition, the surgical techniques and many other local factors, including the insufficient blood supply, the tissue vulnerability, such as local edema and scar on duodenal wall, the length of duodenal stump, and high pressure inside duodenal cavity might influence the healing of duodenal stump and result in DSL (6). So, in order to prevent DSL, reinforcement of duodenal stump is necessary and some reinforcement methods have been applied widely, including barbed suture (12), Lembert suture (13), two half-purse-string sutures (14). However, these methods require multiple stitches and knots, which is rather different for the unexperienced surgeons (12–14). This study was a retrospective, one-arm clinical trial focusing on a new maneuver, single purse-string suture, for reinforcement of duodenal stump in patients harboring gastric adenocarcinoma following laparoscopic radical gastrectomy with Roux-en-Y or BillrothIIreconstruction. The results showed that the morbidity rate was lower compared to laparoscopic assisted distal gastrectomy (morbidity rate 15.2%) in our previous CLASS-01study (15), and there was no incidence of DSL in this research, which proved that single purse-string suture is feasibility and safety.

The laparoscopic duodenal stump reinforcement was thought to be relatively difficult for most even experienced surgeons to perform due to the complexity of duodenal anastomosis, the restriction of sewing angles, and the uncontrollably of knotting strength (16–18). Based on this situation, we proposed a novel reinforcement method, single purse-string suture, which is an easy and effective method to reinforce the duodenal stump, and could avoid DSL to the some extent. There are three key points about this maneuver. First, the interval of sutures is the critical point for this maneuver. The duodenal wall is vulnerable of being grasped, which could easily arouse acute local inflammation and cause local edema and tissue injuries. If the intervals of sutures are too large, the intervals may expand after local edema recedes, which may increase the risk for DSL. Therefore, we positioned our sutures with an interval of 8–10 mm, which can reinforce the duodenal stump well, and does not affect the blood supply of the duodenal stump. Second, if the length of duodenal stump is <1 cm, the single purse-string suture is not recommended. In this case, continuous suture or interrupted suture of duodenal stump is a better choice. Third, this maneuver should be performed by one operator alone. Many surgeons prefer grasping the duodenum or pushing the duodenal stump by the assistant. While, according to our experience, the kernel of controlling the knotting strength is to perform the knotting alone. The purse string suture is satisfying and trustworthy only when the direction and strength of pushing the duodenal stump are synchronous. The knotting balance could be hardly achieved by manipulation of four laparoscopic instruments.

Reinforcement suturing of the staple line after cutting the duodenum has commonly been accepted and performed for prevention of DSL in patients undergoing laparoscopic gastrectomy (1, 8). Many literatures have proved the effectiveness of reinforcement of duodenal stump in laparoscopic gastrectomy with different methods. Sang Yun Kim proved that laparoscopic reinforcement suture on staple-line of duodenal stump using barbed suture can be considered as one of prevention methods of DSL during laparoscopic gastrectomy for gastric cancer (12). Inoue et al. demonstrated the effectiveness of intracorporeal Lembert's sutures in laparoscopic distal gastrectomy receiving Roux-en-Y reconstruction while with no postoperative DSL in 223 patients (13). Ri et al. reported that duodenal stump reinforcement in laparoscopic gastrectomy with Roux-en-Y reconstruction may reduce the risk of DSL development (0.67% vs. 5.71%, *P* <0.001) and minimize its severity (16). In addition to the reinforcement suturing of the staple line, Ojima et al. introduced a new method, reinforced stapling technique, to reinforce the reconstruction after laparoscopic gastrectomy, which is a feasible and safe procedure for gastric cancer with regard to short-term surgical outcomes (19).

There are several limitations of this study. First, this study was a retrospective analysis and the selection biases cannot be totally avoided; Second, this study was a one-arm clinical trial and there was no control group in this study, while, the result is satisfied, and the advantages of this method also can be confirmed according to previous published researches; Third, the number of patients enrolled in this study was small. The feasibility and safety of laparoscopic single purse-string suture for reinforcement of duodenal stump should be confirmed by a prospective randomized controlled multicenter clinical trial with a large sample size in the future.

In conclusion, laparoscopic single purse-string suture for reinforcement of duodenal stump showed its simplicity and efficiency, which could avoid the incidence of DSL to some extent and might improve overall outcomes of patients with gastric cancer receiving laparoscopic radical gastrectomy.

## Data Availability Statement

The raw data supporting the conclusions of this manuscript will be made available by the authors, without undue reservation, to any qualified researcher.

## Ethics Statement

Ethical approval was granted by the Clinical Research Ethics Committee of Zhongshan Hospital of Fudan University (Shanghai, China). Signed informed consent was obtained from all patients for the acquisition and use of anonymized clinical data.

## Author Contributions

HH and FL: conceptualization and writing-review and editing. HH, HL, and BY: formal analysis and resources. HH: investigation and writing-original draft preparation. All the authors have approved the final manuscript.

### Conflict of Interest

The authors declare that the research was conducted in the absence of any commercial or financial relationships that could be construed as a potential conflict of interest.
